# RGDechi-hCit: αvβ3 Selective Pro-Apoptotic Peptide as Potential Carrier for Drug Delivery into Melanoma Metastatic Cells

**DOI:** 10.1371/journal.pone.0106441

**Published:** 2014-09-23

**Authors:** Domenica Capasso, Ivan de Paola, Annamaria Liguoro, Annarita Del Gatto, Sonia Di Gaetano, Daniela Guarnieri, Michele Saviano, Laura Zaccaro

**Affiliations:** 1 Department of Pharmacy, University of Naples “Federico II”, Naples, Italy; 2 Institute of Biostructures and Bioimaging -CNR, Naples, Italy; 3 Diagnostic and Molecular Pharmaceutics -Scarl, Naples, Italy; 4 Center for Advanced Biomaterials for Health Care @ CRIB- Italian Institute of Technology, Naples, Italy; 5 Institute of Crystallography -CNR, Bari, Italy; University of East Anglia, United Kingdom

## Abstract

αvβ3 integrin is an important tumor marker widely expressed on the surface of cancer cells. Recently, we reported some biological features of RGDechi-hCit, an αvβ3 selective peptide antagonist. In the present work, we mainly investigated the pro-apoptotic activity of the molecule and its ability to penetrate the membrane of WM266 cells, human malignant melanoma cells expressing high levels of αvβ3 integrin. For the first time we demonstrated the pro-apoptotic effect and the ability of RGDechi-hCit to enter into cell overexpressing αvβ3 integrin mainly by clathrin- and caveolin-mediated endocytosis. Furthermore, we deepened and confirmed the selectivity, anti-adhesion, and anti-proliferative features of the peptide. Altogether these experiments give insight into the biological behavior of RGDechi-hCit and have important implications for the employment of the peptide as a new selective carrier to deliver drugs into the cell and as a therapeutic and diagnostic tool for metastatic melanoma. Moreover, since the peptide shows a pro-apoptotic effect, a great perspective could be the development of a new class of selective systems containing RGDechi-hCit and pro-apoptotic molecules or other therapeutic agents to attain a synergic action.

## Introduction

The αvβ3 receptor is a member of the integrin family, heterodimeric membrane glycoproteins, with a prominent role in angiogenesis and metastatic dissemination [1], [2]. Interaction between αvβ3 integrin and the extracellular matrix (ECM) proteins has been identified as the most important survival system for nascent vessels, by controlling different cellular functions, including survival, proliferation, migration and apoptosis [3], [4]. Since this integrin is expressed at high levels on the surface of many cancer cells [5], [6], [7] as well as tumor-associated endothelial cells [8], it has become an important target in the development of new anticancer strategies.

Integrin αvβ3 performs its function by interacting with several ECM proteins containing the RGD motif, recognized by membrane-bound adhesion molecules, playing a key role as cell adhesion mediator [4]. Peptides containing this motif show potent anti-adhesion effects, since they compete for the integrin-matrix interaction and show anti-proliferative, antichemotactic and pro-apoptotic effects [9], [10].

In the last twenty years, a large number of αvβ3 antagonists, including antibodies, small molecules, peptidomimetics, and cyclic RGD peptides, have been developed with the aim of selectively inhibiting αvβ3-mediated processes [11], [12], [13]. Most importantly, Kessler and colleagues in 1996 reported the development of cRGDf[N(Me)]V, an αvβ3/αvβ5 antagonist known as Cilengitide [14] that was in phase III clinical study as anti-angiogenic drug for glioblastoma therapy [15], [16], [17], [18]. Unfortunately, very recently (News/Press release from Merck, February 25, 2013) it was announced that the Phase III trial of the investigational integrin inhibitor Cilengitide did not meet its primary endpoint of significantly increasing overall survival when added to the current standard chemoradiotherapy regimen (temozolomide and radiotherapy). Furthermore, neither Cilengitide nor all known antagonists are able to discriminate between αvβ3 and other type of integrins.

Over the last few years, we reported the development of a new and selective peptide named RGDechi-hCit [19]. It proved able to selectively bind to αvβ3 integrin and did not cross-react with αvβ5 and αIIbβ3 in adhesion and competitive binding assays on stably transfected K562 cells expressing αvβ3. This selectivity is a fundamental feature for the design of new systems with reduced side effects and dosage. In agreement with in vitro findings, imaging studies on human glioblastoma U87MG also indicated that RGDechi-hCit allows selective visualization of αvβ3 expression [20]. Furthermore, very recently we showed the ability of RGDechi-hCit to significantly inhibit some intracellular pathways acting as an αvβ3 integrin inhibitor, and its role as an antiangiogenic agent *in vitro* and in *vivo* [21].

In melanomas, a number of studies have shown malignant transformation to be associated with a general up-regulation of cell adhesion molecules [22], [23]. In particular, it is well-known that over-expression of αvβ3 heterodimer is often associated in melanoma with the transition to a metastatic phenotype [24], [25]. Indeed, while in normal epithelial cells the αvβ3 heterodimer is expressed at very low levels or is even absent, it is highly over-expressed in MM (metastatic melanoma) cells, and this increased expression is correlated with the degree of tumor progression.

Importantly, MM is highly resistant to conventional radio and chemotherapies and remains a disease of poor prognosis, with a survival times of a few months. Different chemotherapeutic agents, alone or in combination, have been employed over the last few years resulting, unfortunately, in limited activity with relatively low response rates [26], [27], and providing so far only a small impact on overall survival. As chemoresistance remains a serious concern for melanoma therapy, a great challenge is the development of molecules, to use alone or in combination with other drugs, having the ability to interfere with systems involved in angiogenesis and metastatic dissemination processes and so be remarkably effective in shrinking melanoma tumors.

Very recently [28], we evaluated the *in vitro* antitumor efficacy of RGDechi-hCit peptide on melanoma cell lines differently expressing αvβ3 integrin. The data obtained showed that RGDechi-hCit induces a significant inhibition of proliferation only on the WM266 cell line, in accordance with its very high surface expression of αvβ3.

On the basis of these promising data and taking into account the key role played by integrin αvβ3 in melanoma progression, the aim of this paper was to thoroughly investigate the biological behavior of RGDechi-hCit on the WM266 metastatic cell line to reinforce its potential as an anticancer drug and as carrier for drug delivery. In particular, adhesion, binding, uptake, proliferation and apoptosis studies by flow cytometry and confocal microscopy were performed.

## Materials and Methods

### Peptide Synthesis, Cyclization and Labelling

Polypropylene reaction vessels and sintered polyethylene frits were supplied by Alltech Italy. MeIm, MSNT, TFA and scavengers were purchased from Fluka; NovaSyn TGA resins, coupling reagents and all amino acids were from Novabiochem. DIPEA was purchased from Romil; piperidine, PhSiH_3_ and Pd(PPh_3_)_4_, NBD-Cl and FITC from Sigma-Aldrich.

RGDechi-hCit (c(KRDGe)MDDPGRNPHHocitGPAT-OH) and the scrambled sequence (Ac-KPGRGHNDPDPGHocitDeMHAT-OH) were synthesized in solid phase by Fmoc chemistry basically as previously reported [19], but introducing some synthetic improvements and a different Lys functionalisation procedure [29]. Briefly, assembly of fully protected peptides was carried out manually in a polypropylene reaction vessel fitted with a sintered polyethylene frit using NovasynTGA resin (0.21 mmol/g) and all standard amino acids, except for D-Glu. The first amino acid was bound to the support by treatment with Fmoc-Thr(tbu)-OH (5 equiv)/MSNT (5 equiv)/MeIm (3.75 equiv) in DCM for 3 h. The Fmoc protecting group was removed by treatment with 30% piperidine in DMF (3×10 min). Coupling reactions of the following amino acids were performed by using 10 equiv of Fmoc protected derivative activated in situ with HBTU (9.8 equiv)/HOBt (9.8 equiv)/DIPEA (20 equiv) or COMU or HATU (9.8 equiv)/DIPEA (20 equiv) in DMF for 30 min. The coupling efficiency was assessed by the Kaiser test. Each step was followed by resin washings (3×5 min). For RGDechi-hCit, before the final Fmoc deprotection, selective α-carboxyl deprotection of the D-Glu residue from the allyl group was carried out by treatment of the peptidyl resin with PhSiH_3_ (24 equiv)/Pd(PPh_3_)_4_ (0.25 equiv) in DCM (2×30 min). The cyclisation between αNH of D-Glu and αCO of Lys was performed with PyBop (1.5 equiv)/HOBt (1.5 equiv)/DIPEA (2 equiv) in DMF for 5 h at millimolar pseudo-dilution. For both peptides Fmoc-Lys(ivDde)-OH was employed as the last amino acid with the orthogonal ivDde protecting group in order to selectively remove it onto the resin and carry on the following functionalisation with the NBD-Cl (4-Chloro-7-nitro-1,2,3-benzoxadiazole) or FITC (fluorescein isothiocyanate) ivDde group which was removed from Lys by using a solution of 2% hydrazine in DMF (10×2 min); coupling reactions were performed with 3 equiv of NBD-Cl or FITC and 6 equiv DIPEA in DMF overnight. Finally the peptides were cleaved off the resin and deprotected using a mixture of TFA/H_2_O/EDT/TIS (94∶2.5∶2.5∶1 *v/v/v/v*) for 3 h. The resins were then filtered, and the white solid peptides were obtained by precipitation from cold anhydrous diethyl ether. The crude products were purified by preparative RP-HPLC on the Shimadzu LC-8A system, equipped with an UV-Vis detector SPD-10A using a Phenomenex Jupiter Proteo column (21.2×250 mm; 4 µm; 90 Å) and a linear gradient of H_2_O (0.1% TFA)/CH_3_CN (0.1% TFA) from 5 to 70% of CH_3_CN (0.1% TFA) in 30 min at a flow rate of 20 mL/min. The purified peptides were analysed and characterised by an ESI-LC-MS instrument (ThermoFinnigan) equipped with a diode array detector combined with an electrospray ion source and a quadrupole mass analyzer, using a Phenomenex Jupiter Proteo column (4.60×150 mm; 4 µm; 90 Å) at a flow rate of 0.8 mL/min and the same gradient used for the purification step.

### Cell lines and culture conditions

Human adenocarcinoma cells line (HeLa) (ATCC U.S.) were grown in DMEM supplemented with 10% fetal bovine serum (FBS), 2 mM glutamine, 100 U/mL penicillin and 100 µg/mL streptomycin (Euroclone, Italy). Human metastatic melanoma cells line (WM266), kindly provided by Dr. Gentilcore (IRCCS-Fondazione Pascale, Naples Italy), were grown in RPMI, supplemented with heat-inactivated 10% fetal bovin serum (FBS), 1% glutamine, 100 U/mL penicillin and 100 µg/mL streptomycin. The cells were maintained in humidified air containing 5% CO_2_ at 37°C.

### FACS analysis for αvβ3 integrin

Adherent cells at about 70% confluence were detached using 0.1 mM EDTA in PBS (Sigma Aldrich), centrifuged and suspended in PBS containing 0.2% BSA. Cell aliquots (2.5×10^5^ cells) were treated with primary monoclonal antibody, clone LM609 (Millipore MA, USA) or isotype control (Santa Cruz Biotechnology, Germany), at the same concentrations for 30 min at 4°C. After washing, the cells were incubated with secondary antibody FITC-conjugated (Santa Cruz Biotechnology, Germany), washed and analysed by using a flow cytometer equipped with a 488 nm argon laser (FACScan, Becton Dickinson, USA). A total of 20000 events per sample were collected. Values of fluorescence intensity were obtained from the histogram statistic of CellQuest software.

### Cell adhesion and detachment assays

NUNC MaxiSorp 96 well plates (Dasit Sciences, Italy) were coated overnight at 4°C with vitronectin, fibronectin or collagen type I (Millipore, USA), at 10 µg/mL concentration diluted in PBS, pH 7.4; then a blocking step for nonspecific binding was performed with 1.5% BSA in PBS for 1 h at room temperature. WM266 cells were suspended and mixed in Hank's balanced salt solution (HBSS: 50 mM HEPES, 1 mg/ml BSA, 1 mM CaCl_2_, 1 mM MgCl_2_, 1 mM MnCl_2_, pH 7.4, Sigma Aldrich, Italy) with peptides (50 µM) or anti-αvβ3 antibody (10 µg/mL) for 30 min at 4°C and then plated on pre-coated plates (1.5×10^4^cells/well). Non adherent cells were gently removed by repeated washings. After 1 h of incubation, the adherent cell number was evaluated by crystal violet (Sigma Aldrich, Italy) assay, which correlates optical density with cell number [30]. Briefly, cells were washed with PBS and fixed by adding 10% formalin solution. After 15 min cells were washed with deionized water and stained with 100 µl of 0.1% crystal violet solution in water for 30 min. Excess dye was removed by washing with deionised water and plates were air-dried prior to bound dye solubilisation in 200 µL of 10% acetic acid. The optical density of dye extracts was measured directly in plates at 595 nm (BioRad microplate reader model 680). The mean value ± SE of adherent cells for each treatment was expressed as relative percentage of cell number *vs* cells not treated (control). Statistical differences were determined by Student's t test, unpaired, two-sided. All experiments were performed in triplicate and repeated at least 3 times; a p value less than 0.05 was considered to be significant.

### Cell proliferation assay and apoptosis

WM266 cells were seeded at 4×10^3^ cells/mL in 96-well plates in RPMI 10% FCS. After 24 h the cells were starved (4% FCS in RPMI) for 4 h and incubated with increasing concentrations (10, 25 and 50 µM) of peptides. The proliferation was evaluated after 24 h using crystal violet assay.

The apoptosis assay was analysed on WM266 cells seeded at 2×10^5^ cells/well in a 6 well plate and starved as described above. Next, the cells were incubated at 37°C with different peptides at 50 µM concentration, and apoptosis was analysed after 16 h by staining with annexin V/FITC and Propidium iodine (PI) (eBioscience, USA). Briefly, after incubation, the untreated and treated cells were detached with Accutase solution (eBioscience), harvested and washed with cold PBS. Subsequently, the cells were treated following the manufacturer' instructions. The percentage of cell undergoing apoptosis or necrosis was quantified using a flow cytometer (Becton Dickinson, USA) equipped with Cell Quest software.

### Cell binding and uptake of peptides by FACS

WM266 cells were detached with 0.1 mM EDTA in PBS, washed and incubated with peptides. In detail, 2.5×10^5^ cells were treated with 10 µM NBD-labeled RGDechi-hCit or 10 µM scrambled peptide, in 100 µL of 20 mM CaCl_2_/HBSS at room temperature in slow agitation for 30 min. Then, the cells were washed in 20 mM CaCl_2_/HBSS and resuspended in the same buffer to perform FACS analysis. The NBD-labeled peptides were excited at 465 nm and fluorescence was measured at 530 nm. After the analysis, 5 µL of dithionite (Sigma Aldrich, Italy) stock solution (1 M freshly prepared in 1 M Tris-HCl pH 10) was added to cells at 4°C for 5 min and the fluorescence of internalised peptides was detected by FACS.

### Cellular uptake of peptides by confocal microscopy

WM266 and HeLa cells were seeded on a 35 mm diameter Fluorodish Cell Culture Dish (World Precision Instruments, Inc., Florida) at the concentration of about 5×10^4^ cells/mL. 24 h after seeding, cells were incubated with FITC-RGDechi-hCit and FITC-RGD scrambled at the final concentration of 50 µM in HBSS buffer for 30 min at 37°C. Cells were rinsed twice with PBS to remove peptide excess and fixed with 4% paraformaldehyde at room temperature. Samples were then observed with a confocal microscope (Leica SP5) with a 63× oil immersion objective with a 488 nm wavelength laser line and transmitted light.

### Indirect immunofluorescence

For co- localization experiments, after peptide incubation, cells were first rinsed twice with PBS to remove non-internalised peptide and fixed with paraformaldehyde 4% for 20 min. The cells were washed for 10 min in 10 mM PBS/20 mM glycine, permeabilised with 10 mM PBS/20 mM glycine containing 0.005% saponin for 7 min, and blocked with PBS/20 mM glycine 1% albumin. αvβ3 integrin was performed by using mouse anti-human integrin αvβ3 primary antibodies (Chemicon) and Alexa-fluor 568 goat anti-mouse secondary antibodies (Molecular Probes, Invitrogen). Endocytic vesicles were localised by incubating samples first with mouse anti-clathrin monoclonal (ABR) and rabbit anti-caveolin 1 (Abcam) primary antibodies and then with Alexa-fluor 568 goat anti-mouse and anti-rabbit secondary antibodies (Molecular Probes, Invitrogen), respectively. Lysosomes were localised with rabbit anti-LAMP 2 polyclonal (Abcam) primary antibodies and with 568 goat anti-rabbit secondary antibodies (Molecular Probes, Invitrogen).

All samples were then observed with a confocal microscope with a 63× oil immersion objective. Co-compartimentalisation between the peptide and the endocytic markers was analysed with ImageJ analysis software plugin.

### Inhibition of clathrin- and caveolin-mediated endocytosis

Hypertonic challenge was carried out by incubating the WM266 cells with 0.45 M sucrose for 20 minutes at 37°C [31] in order to inhibit the clathrin-mediated endocytosis. Treatment aimed at inhibiting caveolae-mediated endocytosis was evaluated by incubating cells with filipin at 5 µg/mL for 30 minutes at 37°C. After both treatments, cells were incubated with 50 µM RGDechi-hCit solution in HBSS buffer for 30 minutes at 37°C. Then, cells were washed twice with PBS. Some samples were fixed with paraformaldehyde for the confocal microscopy observations and other ones were lysed with 1% Triton ×100 in PBS for the quantitative analysis. To this aim, the intensity fluorescence of cell lysates was measured at an excitation wavelength of 488 nm by a spectrofluorimeter (Perkinelmer). Results reported as percentage of cellular uptake were normalized with respect to non-treated control cells (expressed as 100%).

## Results

### Synthesis of functionalized RGDechi-hCit and scrambled peptides

Peptides were synthesized by the solid-phase method using Fmoc chemistry. All amino acids were coupled according to the HBTU/HOBt/DIPEA/DMF procedure [32]. To improve the overall yield of RGDechi-hCit over that previously reported (24%) [19], in the case of more difficult reactions a double coupling procedure was performed or more efficient coupling reagents were employed, e.g. COMU or HATU/DIPEA DMF. Before the Fmoc deprotection of Lys^1^, α-carboxyl-selective deprotection of the D-Glu^5^ residue from the allyl group was carried out by the treatment of the peptidyl resin with a solution of PhSiH_3_/Pd(PPh_3_)_4_ in DCM [33]. The cyclisation between the αNH group of Lys^1^ and the αCO group of D-Glu^5^ was performed with PyBop/HOBt/DIPEA [34] in DMF.

For both peptides, Fmoc-Lys(ivDde)-OH was employed as the last amino acid with the orthogonal ivDde protecting group in order to selective remove it onto the resin and carry out the subsequent functionalisation with NBD-Cl or FITC. The ivDde group was removed from Lys by using a solution of 2% hydrazine in DMF. The coupling reactions were performed using NBD-Cl or FITC and DIPEA in DMF overnight. Final deprotection and cleavage from the resin were achieved with TFA and scavengers.

The peptides were purified by preparative RP-HPLC; the purity and identity of the peptides were confirmed by analytical RP-HPLC and ESI-LC-MS mass spectrometry. The used synthetic conditions permitted the increase of the overall yield of the peptide up to 30%. This is a good result considering the chimeric nature of a molecule having both a cycle and linear motif and a lysine residue for its functionalization in view of its specific applications.

### Expression of αvβ3 integrin on human melanoma cells (WM266) and human adenocarcinoma cells (HeLa)

**Figure 1:**
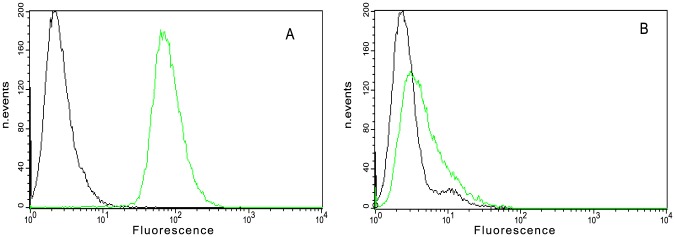
Analysis of expression of αvβ3 integrin in WM266 and HeLa cells by flow cytometry. Pre-confluence cells (WM266 cells (A), HeLa cells(B)) were detached by 0.1 mM EDTA in PBS and incubated with anti αvβ3 antibody (green curve) or isotype (black curve) and then with secondary antibody FITC conjugate.

Evaluation of the expression levels of αvβ3 integrin on the membrane surface of WM266 and HeLa cell lines was performed by flow cytometric analysis using the monoclonal antibody LM609. To maintain surface marker integrity, cells were detached using 0.1 mM EDTA in PBS. Data showed that WM266 cells expressed αvβ3 at high level, while HeLa cells displayed low levels of αvβ3. Specifically, the relative percentage of fluorescence is about 5% on HeLa cells and 85% on WM266 (Figure 1). Therefore we used WM266 and HeLa cell lines as positive and negative controls for αvβ3 integrin expression, respectively.

### Effect of RGDechi-hCit on WM266 adhesion

The influence of RGDechi-hCit on the adhesion of WM266 cells seeded onto vitronectin (a matrix able to recognise both αvβ3 and αvβ5 integrins), fibronectin (recognising αvβ3) or collagen type I (not recognising either αvβ3 or αvβ5 integrins) [35], [36], [37] was investigated in a specific cells adhesion buffer [38] containing fundamental bivalent ions for integrin activity, such as Mg^2+^ or Ca^2+^, to perform the assay under optimal conditions.

**Figure 2:**
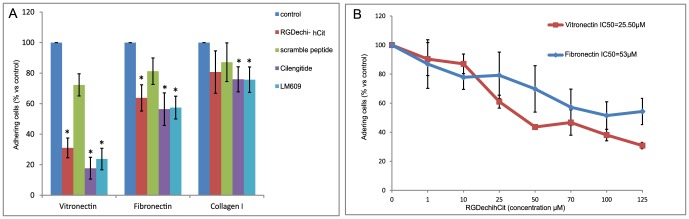
Inhibition of adhesion of WM266 on different extracellular matrix coated plates (A). Cells were pre-incubated with peptides (50 µM) or anti-αvβ3 antibody (10 µg/mL) for 30 min at 4°C and then seeded on extracellular matrix pre-coated plates. Cell adhesion was evaluated after 1 h of incubation using crystal violet reagent. The results are presented as the percentage of adherent cells respect to the control (untreated cells) and are expressed as means ± SE of three independent experiments performed in triplicate. Statistical significance was analyzed using Student's t test, unpaired, two-sided (*p<0.05). RGDechi-hCit dose-effect on WM266 cell adhesion (B). Cells were pre-incubated with increasing concentrations of RGDechi-hCit for 30 min at 4°C and then seeded on vitronectin or fibronectin (10 µg/mL) pre-coated plates at 37°C. The cell adhesion was evaluated after 1 h of incubation using crystal violet reagent. The results are presented as the percentage of adherent cells respect to the control (untreated cells) and are expressed as mean ± DS from three independent experiments performed in triplicate.

In order to prevent receptor internalisation, cells were preincubated at 4°C in adhesion buffer with peptides or monoclonal antibody anti αvβ3 integrin and then seeded into plates previously coated with different matrices. As shown in Figure 2A, RGDechi-hCit decreased the adhesion of WM266 plated onto vitronectin (∼70%) and fibronectin (∼40%), but did not have significant effect on adhesion to collagen type I. Similar data were obtained with cilengitide used as positive control and with the monoclonal antibody LM609 used to demonstrate the specificity of αvβ3 -dependent adhesion.

Incubation of cells with scrambled peptide, used as negative control, did not decrease WM266 adhesion onto matrix proteins. Furthermore, it is worth noting that the inhibition of adhesion by RGDechi-hCit occurred in a concentration-dependent manner giving an IC50 of 25.5 µM and 53 µM for vitronectin and fibronectin, respectively (Figure 2B).

**Figure 3:**
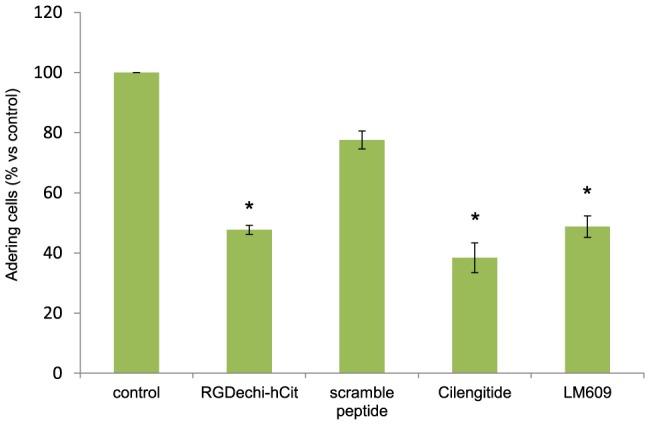
Detachment of WM266 cells from vitronectin coated plates. Cells were plated onto vitronectin (10 µg/mL) pre-coated wells and were allowed to adhere for 1 h. Peptides (50 µM) or anti-αvβ3 antibody (10 µg/mL) were added and the incubation was continued for 2 h. Cell adhesion was determined by crystal violet assay. Results are presented as percentage of adherent cells respect to the control (untreated cells) and are expressed as means ± SE of three independent experiments performed in triplicate. Statistical significance was analyzed using Student's t test, unpaired, two-sided (* p<0.05).

In addition, the effect of RGDechi-hCit on cell detachment from vitronectin was tested 1 h after attachment of cells to vitronectin, the peptide was added and a significant cell detachment was observed (50%); an analogous effect was obtained using Cilengitide (60%) and antibody LM609 (50%), while using a scrambled peptide, no significant effect was observed (Figure 3), further confirming the RGDechi-hCit specific activity on αvβ3-dependent adhesion.

### RGDechi-hCit inhibits proliferation and induces apoptosis in WM266 cells

**Figure 4:**
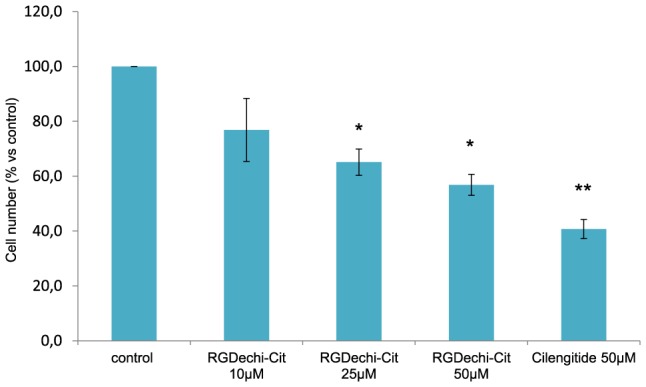
Cytotoxicity assay. WM266 were treated with starvation conditions (4% FCS) for 4 h and then peptides at different concentrations were added and incubated for 24 h. The proliferation was evaluated using crystal violet assay. The results are presented as the percentage of proliferating cells respect to the control (untreated cells) and are expressed as means ± SE of three independent experiments performed in triplicate. Statistical significance was analyzed using Student's t test, unpaired, two-sided (* p<0.05, **p<0.01).

To evaluate cell proliferation, starved WM266 were incubated with Cilengitide (50 µM) or with RGDechi-hCit peptide at different concentrations (10-50 µM) for 24 h. The inhibition of cell proliferation results in a dose-dependent manner. At the higher concentration used, RGDechi-hCit peptide induced a significative inhibition of proliferation (∼45%) in respect to the untreated cells (Figure 4) and comparable to the Cilengitide (∼60%)

**Figure 5:**
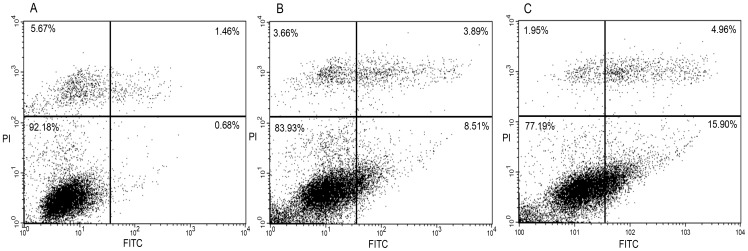
Apoptosis analyses with annexin V-FITC/PI double staining on WM266 cells. Untreated cells (A); cilengitide treated cells (B); RGDechi-hCit treated cells (C). Upper left quadrant: necrotic cells; upper right: advanced apoptotic cells; lower left: viable cells; lower right: early apoptotic cells. These pictures are representative of three independent experiments.

In the same conditions of starvation, after 16 h of treatment with RGDechi-hCit or Cilengitide (both 50 µM), apoptosis was evaluated with annexin V-FITC/PI double staining by flow cytometry analysis. Starved WM266 cells, treated with RGDechi-hCit, exhibited 8.5% early apoptotic cells, 3.9% late apoptotic and 3.7% of necrotic cells; whereas using Cilengitide, 16.0% early apoptotic cells, 5.0% late apoptotic and 2.0% of necrotic cells were obtained (Figure 5). The same experiment conducted on starved cells in the absence of peptides displayed 0.7% early apoptotic cells, 1.4% late apoptotic and 5.6% necrotic cells.

### RGDechi-hCit cellular uptake by flow cytometry analysis and confocal microscopy

The uptake of NBD-RGDechi-hCit into WM266 cells was determined by flow cytometry. The trans-bilayer distribution of NBD-peptides was determined by comparing the fluorescence intensity before and after addition of sodium dithionite, an essentially membrane-impermeant molecule, which irreversibly suppresses the fluorescence of the accessible NBD-moiety localised on the external cell surface [39]. The NBD-RGDechi-hCit (10 µM) was incubated with WM266 cells for 30 min at 37°C and then its quenching by dithionite treatment, was measured. To avoid dithionite diffusion in the biological membranes, this reaction was performed at 4°C. The addition of dithionite determined a strong increase of fluorescence compared to the intrinsic fluorescence of the cells. This result showed that a remarkable percentage of peptide (∼90%) was internalised. In the same experimental conditions, NBD-scrambled peptide was used to exclude the non-specific fluorescence signal (data not show).

To confirm the peptide cell penetration and to collect information about the mechanism of internalisation in WM266 cells, confocal studies were also performed.

**Figure 6:**
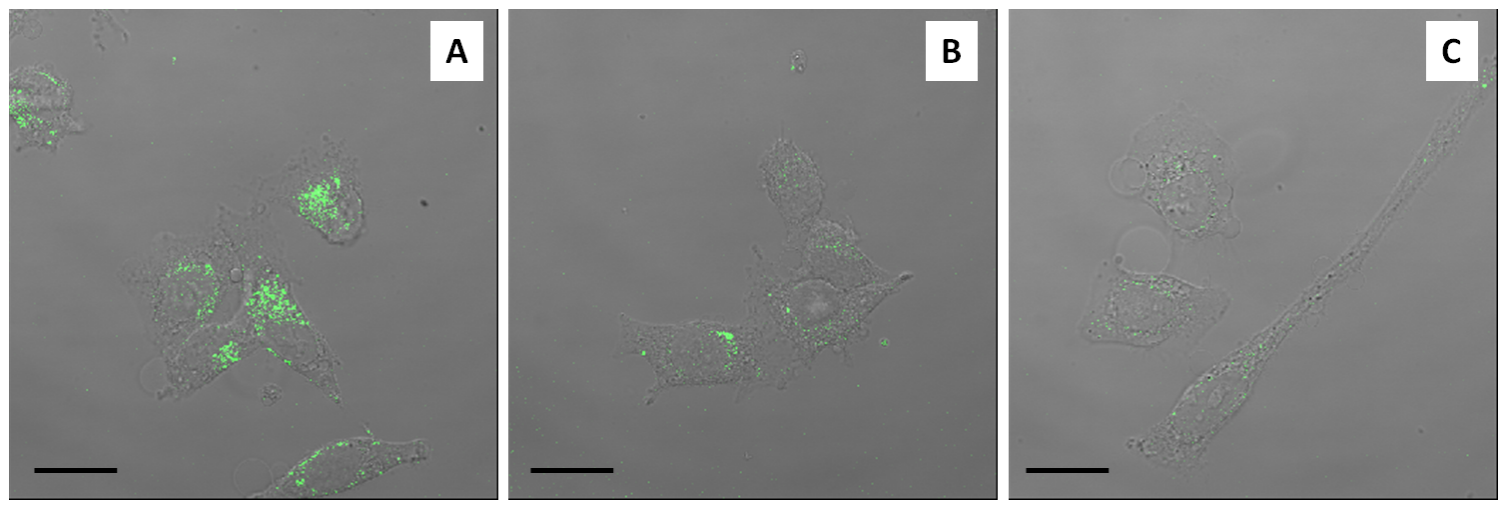
Merge of confocal and transmitted light images of peptide cellular uptake. WM266 cells after 30 min incubation with 50 µM RGDechi-hCit (A) and scrambled peptide(B). HeLa cells after 30 minute incubation with 50 µM RGDechi-hCit (C). Green fluorescence indicates peptides. Magnification bar: 20 µm.

FITC-RGDechi-hCit was efficiently internalised after 30 min of incubation at 37°C (Figure 6A). Indeed, confocal images show that the peptide was localised within cell cytoplasm and mainly distributed into discrete spots suggesting its confinement in vesicular structures. Conversely, no cellular uptake was observed for FITC-scrambled peptide in WM266 cells (Figure 6B) and also for FITC-RGDechi-hCit in HeLa cells (Figure 6C) not expressing αvβ3 integrin receptor.

**Figure 7:**
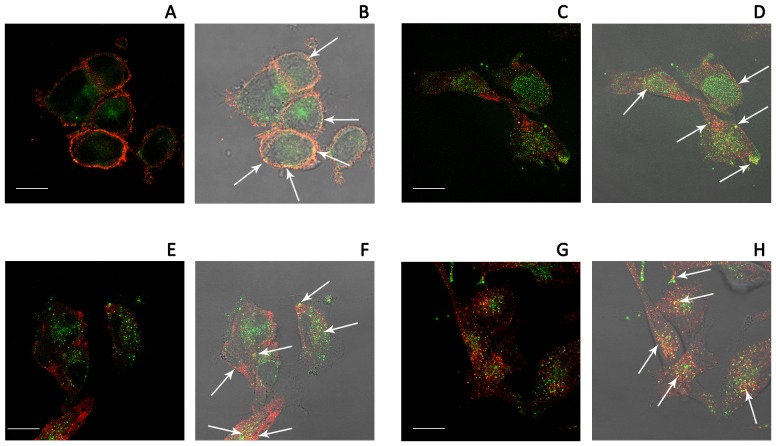
Co-localization areas (white arrows) of RGDechi-hCit peptide (green) with αvβ3 integrin (red)(A), caveolin 1 (red)(B), clathrin (red)(C) and LAMP 2 (red)(D) in WM266 cells after 30 minute incubation. (A, C, E, G) fluorescence images; (B, D, F, H) transmitted and fluorescence image overlapping. Magnification bar: 20 µm.

In order to investigate the mechanism of RGDechi-hCit peptide uptake, co-localisation experiments by using anti-αvβ3 integrin antibody were carried out. Figure 7A shows a partial co-localisation of FITC-RGDechi-hCit with αvβ3 integrin along cell boundaries (white arrows), suggesting a possible interaction between the peptide and the receptor at the cell membrane level.

Endocytosis of αvβ3 integrin was described to occur through clathrin-dependent [40] or caveolae endocytosis [41]. Therefore, to determine whether RGDechi-hCit peptide uptake followed the same endocytic pathways, we performed indirect immunofluorescence analyses using anti-clathrin and anti-caveolin 1 antibodies. Results showed a partial co-compartimentalisation of RGDechi-hCit both with caveolin 1 (Figure 7B) and clathrin (Figure 7C) proteins. A clear co-compartimentalisation of RGDechi-hCit with LAMP2 (Lysosome-Associated Membrane Proteins, whose function is mainly related to the protection, maintenance, and adhesion of the lysosome) was found, indicating an accumulation of this peptide in lysosomes (Figure 7D), as a possible consequence of receptor-mediated endocytic pathway.

**Figure 8:**
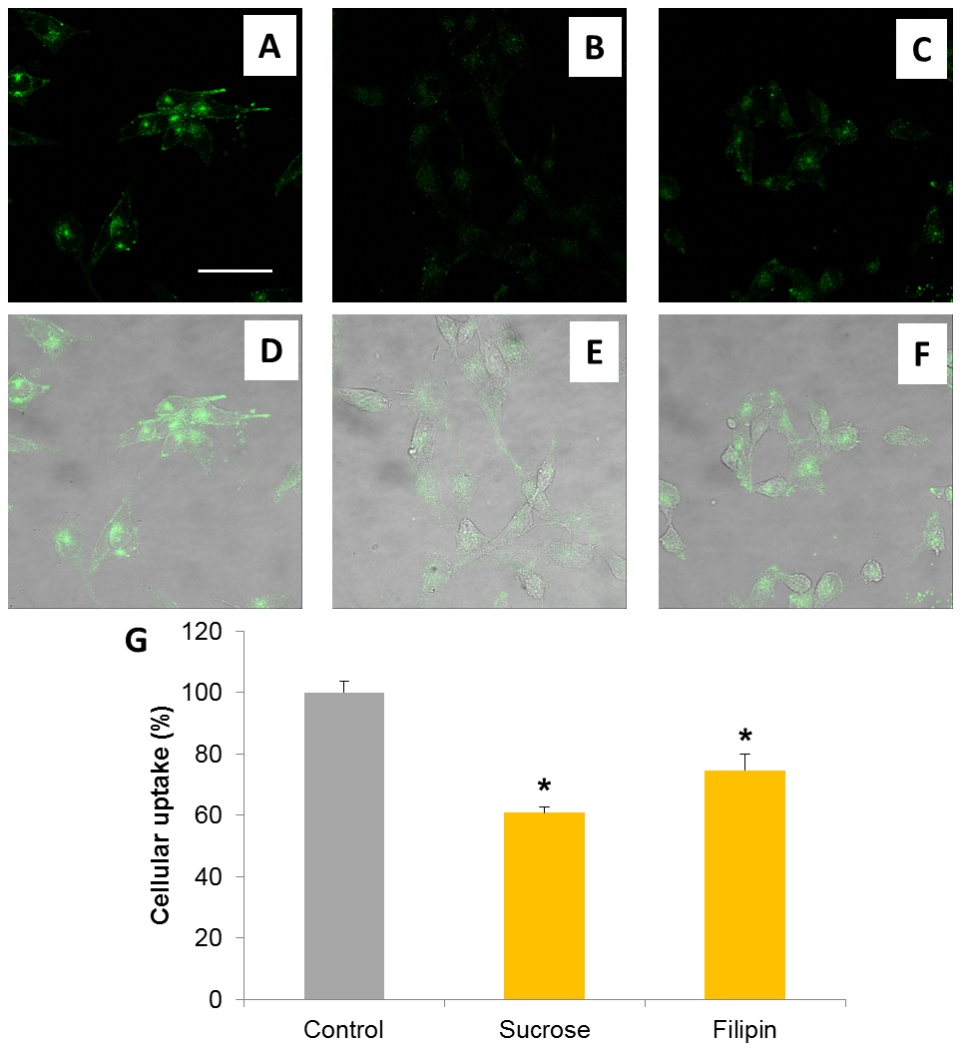
RGDechi cellular uptake inhibition experiments. A–F, Confocal microscope images of WM266 cells after 30 minutes of incubation with 50 µM RGDechi. A and D control non-treated cells; B and E cells treated with 0.45 M sucrose; C and F cells treated with 5 µg/ml filipin. A–C fluorescence images; D–F merge of fluorescence and transmitted light images. Magnification bar 50 µm. G, quantitative analysis of RGDechi cellular uptake by spectrofluorimeter measurements. Data are expressed as mean ± standard deviation (* p<0.05).

To further elucidate the mechanism of RGDechi-hCit peptide uptake, WM266 cells were treated with sucrose and filipin which inhibit clathrin- and caveolin-mediated endocytosis, respectively. Confocal images showed a decrease of intracellular peptide fluorescence after both treatments compared to non-treated cells (Figure 8 A–F). A quantitative analysis indicated a reduction of about 40% and 20% RGDechi-hCit cellular uptake upon sucrose and filipin treatments, respectively (Figure 8 G). These results confirmed that the uptake of RGDechi-hCit peptide partially involves clathrin- and caveolin-mediated endocytic mechanisms.

## Discussion and Conclusion

Several ECM proteins, enclosing the RGD motif, play a crucial role in integrin-mediated cell adhesion and consequently, in cell proliferation, survival and migration [4], [42], [43]. So far a huge variety of RGD peptides and mimetics are reported in the literature as potential candidates for tumor imaging, cell-targeting and therapy [44], [45], [46], [47] thanks to their ability to recognise integrin receptors and eventually to internalise into different cell types, including endothelial and melanoma cells [42], [48], [49], [50], [51]. Many studies have identified marked differences in the surface expression and distribution of integrins in malignant tumor cells [52]. In particular αvβ3 integrin is expressed strongly on the surface of malignant melanoma cells and angiogenic blood vessels, but weakly on pre-neoplastic melanomas and quiescent blood vessels [30], [38]. So a high expression of αvβ3 integrin seems to be correlated to the increase of the metastatic potential of the melanoma cell line [19], [28], [39], [40].

Over the last few years we focused our attention on the study of the biological features of a highly selective chimeric αvβ3 antagonist named RGDechi-hCit. Very recently [28], we reported some data on *in vitro* antitumor efficacy of RGDechi-hCit on melanoma cell lines differently expressing αvβ3 integrin. In particular, the data showed that the peptide induces a significant inhibition of proliferation only on the WM266 cell line, in accordance with its very high surface expression of αvβ3.

Here we mainly investigated the pro-apoptotic activity of RGDechi-hCit peptide and its ability to penetrate the WM266 cell membrane. Simultaneously we deepened the selectivity, anti-adhesion, and anti-proliferative features of the peptide. Interestingly, for the first time we demonstrated the ability of RGDechi-hCit to enter cells overexpressing αvβ3 integrin. This exciting result opens the way to consider this peptide as a new potential carrier to deliver drugs into the cell also confirming its selectivity.

In detail, to reinforce the evidence regarding the receptor selectivity of the molecule, we investigated the adhesion effect of RGDechi-hCit on WM266 cells, using vitronectin, fibronectin and collagen I as different matrix proteins. Remarkably, we observed that peptide activity depends on the matrix used; it evidently inhibits cell adhesion to vitronectin and somewhat to fibronectin in contrast to the effect on collagen I, which does not interact with αv integrin and protects cells from the RGDechi-hCit anti-adhesion activity. The higher effect observed on cells seeded on vitronectin with respect to fibronectin is very likely to be ascribed to the different level of receptor expression on the WM266 cell surface, where the presence of the αvβ3 receptor dominates over that of αvβ5 (αvβ3:αvβ5, 10∶1 ratio, data not shown).

Since vitronectin interacts with αvβ3 as well as αvβ5 receptors while fibronectin interacts with α5β1, αIIbβ3 and αvβ3, we have strong reasons to believe that the different behavior of RGDechi-hCit on the various matrices is due to the capability of the WM266 cells to bind fibronectin most efficiently, ascribable to the presence of other integrins not directly interacting with RGDechi-hCit. The inhibition of adhesion by RGDechi-hCit occurs in a concentration-dependent manner for vitronectin and fibronectin and the ability to detach adherent cells from vitronectin is very similar to that of the anti-αvβ3 antibody, reinforcing our findings that the action of the peptide is integrin specific. Additional experiments confirmed our previous data [28] that RGDechi-hCit significantly inhibits the growth of melanoma cells. The anti-proliferative effect of the peptide on cancer cells is comparable to Cilengitide used as positive control and to other RGD–based systems reported in the literature [9], [10].

Interestingly, studies by FACS indicated that RGDechi-hCit is also able to induce apoptosis in a similar way to Cilengitide on other cell lines [53] and in accordance with other reports indicating that cells detached by treatment with RGD molecules go on to apoptosis. This outcome enhances the value of the RGDechi-hCit peptide even if the principal criterion for its success was the evidence of its cell entry ability. For this reason, particular attention was paid to the investigation of the mechanism of internalisation both by flow cytometric and confocal microscopy studies. Generally, RGD-based peptides are able to cross the cell membrane both by passive diffusion [54], [55] and receptor specific mechanism. Here we reported that our peptide is able to enter cells overexpressing αvβ3 integrin and shows a partial co-localisation with anti-αvβ3 integrin antibody, suggesting very likely an αvβ3 receptor-mediated uptake.

Actually, indirect immunofluorescence analysis highlighted a partial co-compartimentalisation of RGDechi-hCit both with caveolin 1 and clathrin proteins. This result was confirmed by using inhibitors of clathrin- and caveolin-mediated endocytosis which induce a clear reduction of peptide cellular uptake. What is more, an evident co-compartimentalisation of RGDechi-hCit with LAMP2 was observed by the accumulation of peptide in lysosome, clear consequence of a receptor-mediated endocytic pathway. Altogether these findings strongly suggest the uptake of RGDechi-hCit occurs mainly through the internalisation of αvβ3 receptor *via* an endocytic pathway. Further investigations will be necessary to confirm such data.

The observed uptake poses the question regarding the mechanism of apoptosis induced by RGDechi-hCit. Studies on RGD peptides have led to several models of antagonist-induced cell death including a type of caspase-dependent apoptosis known as anoikis [42], [56] due to the cells loosing adherence and subsequent detachment caused by interaction with integrins. In our case, the apoptosis could be ascribed both to anoikis and/or to activation of an intracellular pathway consequent to the peptide entrance into the cell. Further studies will be needed to address likely intracellular targets mediating the apoptosis.

All results here reported give some convincing evidence that RGDechi-hCit is able to penetrate cells mainly by αvβ3 integrin-dependent endocytosis and could be considered a novel carrier to deliver selectively drugs into the cell. What is more, we discovered that the peptide displays an apoptotic effect *per se*. This consideration and the previous data on the ability to inhibit some intracellular pathways [21], suggest that RGDechi-hCit can be potentially considered a true αvβ3 integrin inhibitor.

In conclusion, a great challenge to evaluate the achievable application of RGDechi-hCit molecule in vivo is the development of new selective systems containing both our peptide and pro-apoptotic molecules or different therapeutic agents to induce a synergic action.
